# A nutritionally complete pollen-replacing diet protects honeybee colonies during stressful commercial pollination—requirement for isofucosterol

**DOI:** 10.1098/rspb.2024.3078

**Published:** 2025-04-16

**Authors:** Thierry Bogaert, Taylor Reams, Isabelle Maillet, Kelly Kulhanek, Maarten Duyck, Frank Eertmans, Anne Marie Fauvel, Brandon Hopkins, Jan Bogaert

**Affiliations:** ^1^ Apix Biosciences, Wingene 8750, Belgium; ^2^ Department of Entomology, Washington State University, Pullman, WA 99164-6382, USA; ^3^ Bee Informed Partnership, College Park, MD 20742-0001, USA

**Keywords:** honeybees, bee nutrition, sterol, diet, pollination, colony collapse disorder

## Abstract

A steady supply of nutritionally adequate pollen from diverse flower sources is crucial for honeybee colonies. However, climate instability, large-scale agriculture and the loss of flower-rich landscapes have made this supply scarce and unpredictable, threatening both apiculture and sustainable crop pollination. We developed a nutritionally complete pollen-replacing diet that supports continuous brood production from May to October in colonies without access to pollen. Omitting isofucosterol, the third most abundant sterol in honeybees, causes significant reductions in brood production and neuromuscular dysfunction in adults, identifying isofucosterol as a critical micronutrient. In contrast, omitting 24-methylene cholesterol—the most abundant honeybee sterol—does not significantly affect brood production, and surprisingly, bees remain viable without it. Colonies fed a commercial diet severely declined in brood production after 36 days and died out. In a season-long experiment investigating the commercial pollination of blueberry and sunflower fields, a treatment group fed the complete diet overcame the detrimental effects of nutritional stress, unlike colonies in ‘No Diet’ and ‘Commercial Diet’ groups. This study suggests that feeding a complete, pollen-replacing diet to nutritionally stressed colonies can address the root causes of honeybees' growing nutritional deficiencies, supporting their health and their vital pollination services.

## 1. Introduction

Honeybees (*Apis mellifera*) play a vital role in global ecosystems and agricultural productivity as essential pollinators [1]. Worldwide, around 70% of crop plants accounting for 35% of production volume rely on pollination by insects to some degree [2–4]. Their nutritional status is intricately linked to colony health, resilience against environmental stressors and the stability of pollination services. While nectar and pollen provide the primary nutritional sources for honeybees, these natural provisions can be subject to seasonal scarcity, geographical limitations and anthropogenic influences such as habitat loss and pesticide exposure [5]. In this regard, there is a strong demand for proper supplemental diets to maximize colony growth and safeguard bee health. Despite honeybees’ critical role in agriculture, the apiculture industry lags behind other important domesticated species (porcine, poultry, aquaculture, bovine etc.) in the ability to provide them with a well-defined ‘complete’ diet.

While some macronutrients have been well studied in honeybee nutrition, sterols have recently received particular attention (reviewed in [6]). Sterols are essential nutrients for honeybees, playing a critical role in their growth, development and overall health. Honeybees are sterol auxotrophs and unlike most insects they are unable to dealkylate phytosterols into cholesterol [7,8]. Few plants produce pollen that meets all nutritional requirements of honeybees. As such, honeybees must forage selectively from a diversity of flowers to fulfill their nutritional needs [5,9–14].

Honeybees demonstrate flexibility in their utilization of sterols, but the exact nature of this adaptability requires further investigation. The concentration and composition of sterols in pollen vary widely depending on the plant species [15]. Sterols represent 0.2–0.4% of the bodyweight of honeybees, all of which are derived from pollen. More than ninety percent of their sterol composition comprises six sterols. The relative composition of these six sterols varies widely in honeybees and is largely proportional to their relative ratios in the pollen consumed [16]. 24-methylenecholesterol (24MC), beta-sitosterol and isofucosterol (trans−24-ethylidenecholesterol, 24EC) can be found selectively enriched in honeybee tissue if the level of these sterols is low in the pollen consumed [16]. Therefore, there is clearly some level of functional interchangeability between the sterols. However, the degree of interchangeability of the six sterols has not been experimentally established to date, nor whether any of the sterols are essential and have minimum concentration thresholds [17].

While some sterols have clearly defined roles in honeybee physiology, the functions of others remain unclear. Cholesterol (0.4–10.0% of total sterols in honeybees) and campesterol (5–10%) are proposed to be essential because they are the precursors of the hormones 20-hydroxyecdysone and makisterone A, respectively. Beta-sitosterol (10–48%), isofucosterol (13–42%) and stigmasterol (1–10%) have no specific assigned biochemical or physiological roles [16,18]. Beta-sitosterol and stigmasterol when fed as sole sterols to honeybee colonies do not support brood production as well as cholesterol and 24MC [19]. To date, no nutritional experiments in honeybees have been reported with artificial diets containing multiple defined phytosterols or with isofucosterol.

24-methylenecholesterol (24MC) appears to be a particularly crucial sterol for honeybees, impacting brain function and overall colony health. It is the most abundant sterol in all tissues (40–60%) and a structural component of the MRJP1-apisimin−24-methylenecholesterol complex, of which MRJP1 has been shown to be associated with brain function [20–22]. 24MC is considered to be ‘the key sterol’ for honeybees and a critical micronutrient ([16,23] reviewed in [6,24]). Previous research shows that 24MC added to an otherwise sterol-free artificial diet can be beneficial to colony growth [19] or caged honeybee health and survival [23]. A feeding study with ^13^C-labelled 24MC incorporated in an otherwise sterol-free honeybee diet demonstrated that honeybees isolated in cages completely replace their endogenous 24MC every three to four weeks and (assuming 24MC is a critical micronutrient) suggested that, to sustain normal brood rearing, colonies must not have gaps of more than three to four weeks in consuming food containing 24MC [25]. Svoboda *et al*. [16] showed that the total percentage compositions of 24MC, isofucosterol (24EC) and beta-sitosterol can be higher in pupae and adult bees than in the pollen they feed on, indicating the existence of a physiological mechanism to regulate the relative ratios of these sterols in honeybees.

We conducted two long-term feeding experiments (electronic supplementary material, figure S1) as follows.

Experiment 1: This experiment, conducted in tented enclosures, examined the impact on colony performance over a period of 108 days of deleting 24MC or isofucosterol from an otherwise complete diet containing multiple sterols over a period of 108 days.

Experiment 2: This experiment assessed the impact of the complete diet from Experiment 1 on nutritionally stressed colonies under field conditions. Colonies were deployed in commercial pollination services throughout the summer, pollinating blueberry and sunflower crops with intermittent storage in a holding yard—an environment known for low pollen flow and nutritional deficiency.

## 2. Methods

### (a) Experimental diets

We formulated a diet that includes the nutrients that honeybees require (see reviews by [14,26,27]) and added the six sterols in ratios and concentrations similar to those found in naturally foraging honeybees, at our research station in Belgium (Complete Diet A). To test the hypothesis that isofucosterol and 24MC are essential nutrients for honeybees, we created two deficient diets, omitting either isofucosterol or 24MC (Diet B and Diet C, respectively). To keep the total concentrations of sterols in the diets constant we proportionally adjusted the concentration of the other five sterols in the diets. Samples of ingredients of the basal diet and sterol samples were analysed for sterol composition using gas chromatography–mass spectroscopy (GC–MS, [15]) and the sterol concentrations of the diets were calculated (electronic supplementary material, table S1). The full non-sterol composition of the diets is confidential information of APIX Biosciences NV and is not disclosed; however, requests for the diet for research purposes will be considered. Diet D is an internal reference diet used to compare between experiments and trial seasons. Two widely used commercial protein supplement diets were used as baseline: Diet E (Experiment 1; available in EU and USA) and Diet COMP (Experiment 2; available in the USA). Experiment 2 also included a treatment wherein the bees received no supplementary protein patty (NF) and were fully dependent on foraging pollen.

### (b) Experiment 1: Tent study with no access to external food

#### (i) Experiment set-up

We set up an assay with tent-contained colonies with no access to external food. These colonies were fed pollen-free diets (treatments) with differing sterol compositions but an identical total sterol concentration, in the range of that found in pollen and honeybees (electronic supplementary material, table S1). The diets were formulated in the form of a patty (standard beekeeping) and placed on top of the brood frames. The experiment included a treatment group fed a commercially available protein supplement as a reference diet.

Six small hives (‘Mini plus’, nucleus mating hives) per diet were populated with 800−850 ml bees (approximately 2000 bees) and a queen, according to standard beekeeping practices. The queens were freshly mated sister queens of identical age and genetic background (*Apis mellifera ligustica*). These colonies were kept outside to allow queen-mating flights before the start of the study. They were distributed over adjacent tent enclosures of 2 m × 4.5 m × 3 m with a concrete floor, located in Wingene, Belgium, once it was confirmed that all queens had started laying eggs (day 0 = 23 May). Each tent contained three colonies, and two tents were allocated per treatment group (electronic supplementary material, figure S2). As such, all three colonies within the same tent received the same diet throughout the course of the study. Varroa was monitored by examining the photographs of the frames for mites, and Nosema by monitoring for brown diarrhea in combs and outside the hive. Varroa and Nosema levels in the colonies were imperceptible in all treatments.

On day 0, only traces of pollen and bee bread were present in the hives. The diets were provided to each colony every 6 days by laying one or more patties of 50 grams on top of the brood frames and removing leftover diet as per standard beekeeping practice. The dose was adapted over the course of the experiment to account for colony growth. All colonies received an equal amount of their respective diet. The bees had *ad libitum* access to water and 50% sucrose sugar syrup in each enclosure. Water and sugar syrup were refreshed on a weekly basis.

#### (ii) Feed consumption and brood production measurements

Food consumption was measured every 6 days by weighing the leftover diets of each colony. All brood frames were photographed every 12 days over 108 days, and the capped brood cells were manually counted. Since the capped brood stage lasts 12 days, all capped brood cells produced in a colony were counted once and only once. The capped brood observed on day 12 is derived from nutrition collected prior to day 0 (D0) and was not included in the trial data analysis. Accounting for the 21-day ‘egg to emerged honeybee’ cycle of honeybees, all capped brood present from D24 onwards was produced from consumed artificial diets. Any colonies with queens that stopped laying within the first 24 days were deemed to have queens that were not accepted at the creation of the colony and were removed from the experiment.

#### (iii) Bee behaviour assessment

Phenotypic analysis was performed on days 98−99 and 115. These were sunny days with temperatures above 22 °C where normal flight activity was observed. Hives were opened and a populated frame was lifted from the colony. Observers were not blinded to the diet treatment. First, the bees on the frame were observed without disturbing them and their activity was noted. Then, we gently disturbed the bees with the tip of a pen and brushed them downward, observing their reactivity (crawling upward on the frame), behaviour and flight. The observers scored visually for the following phenotypes: lethargy, shakiness and incoordination, weak reaction time, slow movement and unresponsiveness of the bees on the frame to disturbance with a pen. Phenotypes were recorded as absent (−), present (+), more pronounced (++) and severe (+++). All assays were made by the same two observers with previous experience in behavioural studies in other insect models. The behaviour of the colonies kept outside next to the tents served as reference for normal behaviour.

#### (iv) Analysis of sterols

We sampled 15−25 nurse bees (bees sampled near the eggs and brood) per colony on D84 and D134 in a subset of the colonies. No samples were taken prior to D84 so as not to disturb the growth of the colonies. Each sample of 15−25 bees was split into three subsamples of 5−8 bees. The sterol concentration and composition of the 5−8 honeybees in each subsample were determined. Bees were collected and flash-frozen on dry ice and stored at −30 C. The bees were then freeze-dried and homogenized/dispersed into GCTU buffer [28,29]. The sterols were extracted using organic solvents and data were collected using liquid chromatography mass spectrometry [15,28,29], resulting in three replicate measurements per colony (numbered 1, 2, 3).

On D134 of the experiment, pupae were also dissected out of capped brood from Diet A, B and C treatments. Frames with capped brood were removed from colonies fed Diets A, B and C and placed in separate boxes in an incubator at 34°C. The bees that emerged out of the capped brood cells were counted daily until D154.

#### (v) Statistical methods

The main objective of the statistical analysis was to compare treatment effects on counts and cumulative counts of capped brood cells over the course of the experiment. Only data starting from D24 were considered to allow the colonies to stabilize, to allow for depletion of food reserves in the hives and to measure only brood produced from the diets (i.e. not brood produced from pollen reserves or from sterol pools in the colonies).

Colony-specific changes in capped brood cells were compared between four treatments. Treatment and time and their interaction were used as fixed factors and colonies as random effects in a linear mixed effects model (LMEM).

All analyses were performed in the R v. 4.3.1 [30] using the lme4 package [31]. Systematic departures from the normality assumptions were verified using QQ plots; scatterplots of the residuals versus the predictions were used to check for systematic departures from the mean model. A value of *p* < 0.05 was considered statistically significant.

The colony-specific effect of the treatment on the cumulative counts of capped brood cells was investigated using a LMEM. Model assumptions were not met for the untransformed data (non-normal residuals and non-constant variance of the residuals); however, after logarithmic transformation the assumptions underlying the model were met.

Additional information on the statistical methods is available in the electronic supplementary material.

### (c) Experiment 2: Field study with serial stressors

#### (i) Description

In the spring of 2023, 64 colonies were established in Othello Washington (USA), by installing 1.36 kg (3 lbs) bee packages into one 8-frame hive body box with provided honey frame and kept on standard 8-frame hive pallets. The sister queens from each package were caged and were allowed to be released by the worker bees. The newly established colonies were divided into four groups: Diet A, Diet D, COMP and NF, with each group having 16 colonies at the start.

On 12 May (day 0; D0), the colonies had a pre-assessment feeding and on D5 all hives were moved from the holding yard to commercial blueberry fields in Western Washington State. On D41 they were moved back to the holding yard and from there on D75 to commercial sunflower seed production fields. On D104, they were returned to the holding yard. Hive management followed standard Washington State University beekeeping practices and additional hive boxes were added as needed on an individual hive level.

Assessments were performed on days 11, 25, 41, 68, 106 and 133. The number of frames of bees was recorded visually and the area of capped brood was measured using a 5 cm^2^ grid. The queen status was also recorded, mite levels were measured, and adult bee samples were collected for further molecular analysis (data not shown).

Colonies were fed on days 0, 11, 25, 41, 68, 83 and 95. The diets were formulated in the form of a patty and placed on top of the brood frames (standard beekeeping practice). Any remaining feed was collected, and new feed was given to the colonies for their corresponding diets.

All patties weighed roughly 12 oz, and COMP patties were reduced to match this size.

In feeding events prior to 1 July, all colonies were given one patty. In feeding events after 1 July, colonies that had no remaining patty from the previous feeding were given two patties, and all other colonies were given one patty.

We excluded any colonies with signs of queen failure or non-acceptance and diseases prior to D41, which we ascribed to failures in hive set-up.

The blueberry fields were located in North-Western Washington State, near the town of Mount Vernon, WA. The sunflower pollination fields and the holding yard were both located in Eastern Washington State, near the town of Othello.

#### (ii) Statistical methods for differences in frames of bees and frames of capped brood

Statistical analyses followed the same approach as per the tent experiment. An LMEM was fitted to the data. Treatment, time and their interaction were used as fixed factors and hives as random effects. Additional information on the statistical methods is available in the electronic supplementary material.

### (d) Animal Welfare Regulations and field studies

Honeybees do not fall under animal welfare regulations in both Belgium and the USA. The diets were produced in APIX Biosciences’ pilot animal feed plant, which is FAVV (Belgian Federal Agency for the Safety of the Food Chain) registered and compliant. Field studies were compliant with US and Washington State University regulations.

## 3. Results

### (a) Effect of deleting 24MC or isofucosterol from a nutritionally complete artificial diet (Experiment 1)

#### (i) Effect on feed intake, brood production and behaviour

**Figure 1.**
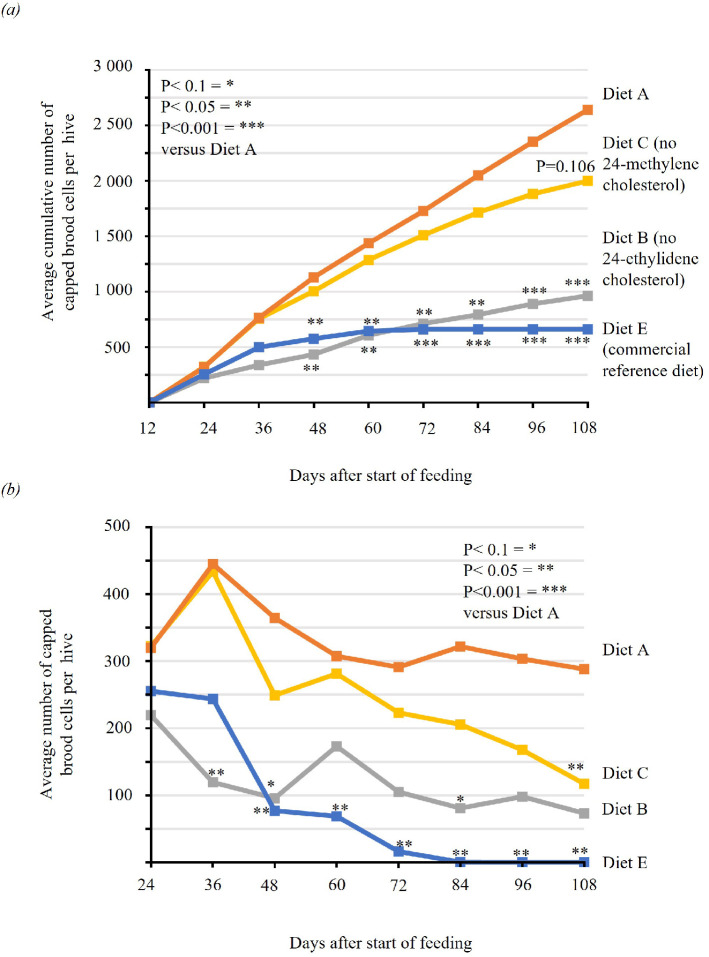
Experiment 1: (*a*) Average cumulative number of capped brood cells for each treatment at each assessment date. (*b*) Average count of capped brood cells for each treatment at each assessment date. Diet A contains all six major sterols: Diet B ( = Diet A minus isofucosterol), Diet C ( = Diet A minus 24MC) and Diet E (a widely used commercial protein supplement). *P* values represent the statistical difference between treatment and Diet A at each time point (linear mixed effects model, details in §2).

Diet A, which contains all six sterols, performed best. Colonies fed exclusively on this diet stably produced brood in the tented enclosures for at least 108 days (figure 1). With an estimated average life span of a worker bee of *ca* 6 weeks [32–34], this 15-week duration represents two renewals of all worker bees in a colony, 9 consecutive brood cycles of 12 days and an estimated *ca* 5 renewals of the 24MC pool in honeybees [23]. The bees were phenotypically wild-type in the enclosure and colony throughout this period (electronic supplementary material, table S2).

Colonies fed the isofucosterol-depleted feed (Diet B) had significantly reduced capped brood production across the 108-day time series. This significantly (P=0.05) reduced brood production compared to Diet A from Day 36 onwards (P< 0.05). At each time-point, colonies fed Diet B produced on average only 36% of the capped brood produced by colonies fed the complete diet. Additionally, the honeybees in colonies fed Diet B exhibited impaired movement and neuromuscular coordination on days 98/99 and day 115.

**Figure 2.**
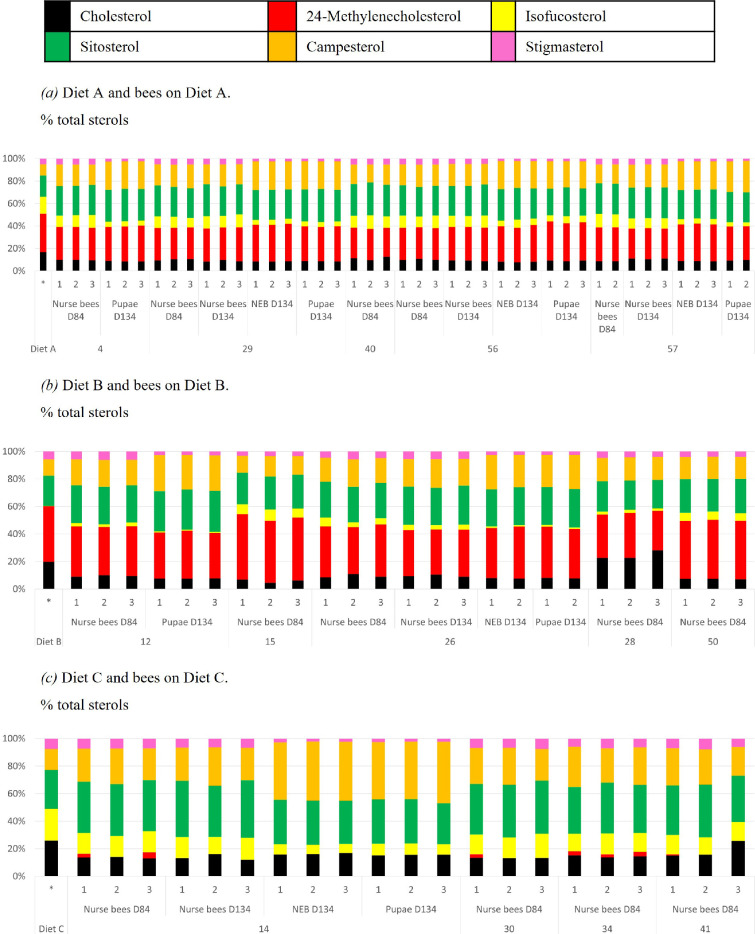
The sterol profiles of the Diets (leftmost bars) and honeybees (next multiple bars) (Experiment 1). Sterol analyses were performed on samples collected on D84 (nurse bees) or D134 (nurse bees, pupae and bees newly emerged from capped brood (NEB), without access to food). 1, 2, 3 are replicates of 5−8 bees each; the numbers at the bottom are the colony codes in the tent experiment. Full data are available in electronic supplementary material, figure S6 and table S11.

Colonies fed the 24MC-depleted feed (Diet C) showed a perceptible decline in capped brood production starting at day 36 and continuing across the 108-day time series compared with colonies fed Diet A. The decline in Diet C continued through until the end at 108 days, whereas brood production in Diet A levelled off and ended with brood production levels similar to that of the start of the experiment (figure 2*b*). However, Diet C was not statistically different from Diet A at 108 days (*p*-value = 0.106). Furthermore, the honeybees in colonies raised for 98/99 and 115 days on Diet C exhibited slower but otherwise normal movement compared with wild type.

Brood production in colonies fed a commercial protein supplement diet (Diet E) was reduced below the sustainable level to replace lost adults. By D48, these colonies produced on average 21% of the brood produced by Diet A and were essentially without brood on Day 72. This indicates that on this diet the bee colonies started lacking one or more specific nutrients after 36 days. The honeybees in colonies raised for 98/99 and 115 days on Diet E were uncoordinated and lethargic, exhibiting the most severe neuromuscular phenotype of all treatments.

All diets were equally well consumed until Day 36. Thereafter, as nutrient depletion led to reduced brood, the amount of feed consumed (electronic supplementary material, table S7) became, as expected, proportional to the amount of brood produced (electronic supplementary material, table S8). The feed conversion ratio of the different diets is shown in electronic supplementary material, table S3.

The emerging rate from incubated D134 capped brood was 100% for all three diets: 181 emerged bees out of 181 capped brood cells for Diet A (3 frames from 3 hives), 22/22 and 33/33 for, respectively, Diet B and Diet C (1 frame from 1 hive each). An emergence rate for Diet E cannot be reported as none of the colonies had any capped brood on day 108. The emerged bees were morphologically normal (no stunting nor wing defects) and were as mobile in the incubator as control bees from a free foraging colony that emerged in parallel.

#### (ii) Effect on the sterol profile of the honeybees

The results of the sterol analysis of honeybee-fed Diets A, B and C are shown in figure 2 (and electronic supplementary material, figure S6, tables S4 and S11) and show that the sterol profiles of the honeybees reflected the profile of the diets they were fed.

The sterol distributions in Diet A (electronic supplementary material, table S1) and in the honeybees fed Diet A for 84 or 134 days) were very consistent across colonies and replicates and were similar to those of honeybees foraging naturally on pollen ([16] and data not shown). Beta-sitosterol and campesterol were enriched and 24MC, isofucosterol and cholesterol were reduced, compared with the sterol profile of Diet A.

Pupae and newly emerged bees contain only the sterols deposited in the egg or fed to them by nurse bees. Pupae and newly emerged bees from colonies fed exclusively Diet A contained 5% isofucosterol of sterols, versus respectively 11% and 10% isofucosterol of total sterols in nurse bees collected on D84 and D134, respectively. The percentages of cholesterol, 24-methylenecholesterol, sitosterol and stigmasterol of total sterols in pupae or newly emerged bees fed Diet A were similar and that of campesterol was higher compared with nurse bees collected on D84 and D134.

The average total sterol concentration in pupae and newly emerged bees from Diet A treatment was 2486 µg total sterol g^–1^ bee DW versus 4018 and 4420 µg total sterol g^–1^ bee DW in nurse bees sampled on D84 and D134, respectively. The average isofucosterol content in pupae and newly emerged bees from colonies fed Diet A was also lower than in nurse bees collected on D84 and D134 (120, 427 and 340 µg g^–1^ bee DW, respectively). Their body sterol profile was reduced in isofucosterol and higher in campesterol compared with nurse bees.

After 84 days on the 24MC-depleted Diet C, the bees showed an average concentration of 64 µg 24MC g^–1^ bee DW (18-fold reduced versus bees fed Diet A) versus 1141 on Diet A and 1686 on Diet B. The 24MC percentage of total sterols was on average 2% (14-fold reduced versus bees fed Diet A in the colonies fed Diet C, compared with 28% in bees fed on Diet A and 38% in bees fed on Diet B.

Fourteen out of 21 samples of nurse bees fed on Diet C collected on D84 and D134 or of honeybees newly emerged from capped brood collected on D134 contained no 24MC. This shows that the colonies were depleted from 24MC in the experiment and, surprisingly, that honeybees without 24MC in their tissues can be morphologically normal and viable. The data also imply that pupae and newly emerged bees were produced without the contribution of 24MC from the worker jelly produced by the nurse bees feeding these.

Honeybees from colonies fed the isofucosterol-depleted Diet B for 84 days had on average 161 µg isofucosterol per gram bee DW versus 427 in bees fed Diet A and 815 in bees fed Diet C. In percentage of total sterols, this is 4% isofucosterol of total sterols in bees fed Diet B compared with 15% in the feed (in Diet A), 11% in bees from colonies fed Diet A and 15% in bees from colonies fed Diet C.

Isofucosterol was reduced 2.7-fold in mg isofucosterol g^–1^ bee tissue and in the percentage of total sterols in honeybees fed Diet B (sampled on D84) compared with bees fed Diet A. However, 24MC was reduced 17-fold in mg 24MC g^–1^ bee tissue and 14-fold in percentage of total sterols in honeybees fed Diet C (sampled on D84) versus bees fed Diet A. Despite the lower level of depletion (or higher level of retention) of isofucosterol in the bees compared with the depletion of 24-methylenecholesterol, isofucosterol depletion had a much more severe effect on brood production, colony collapse and neuromuscular function than 24MC depletion.

### (b) Supplementary feeding with an artificial pollen-replacing diet mitigates stress during serial blueberry and sunflower pollination with intermittent holding yard storage and avoids colony collapse (Experiment 2)

**Figure 3.**
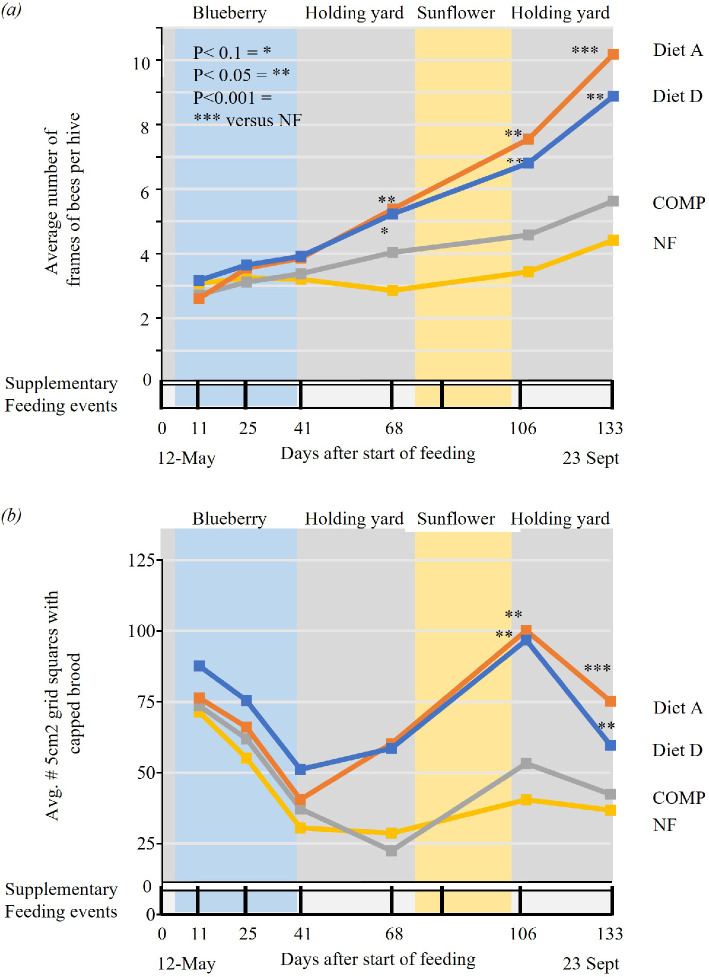
Diet A mitigates the serial stresses that colonies experience in a typical commercial summer pollination cycle (Experiment 2). Over a 133-day period, 16 colonies per treatment group were serially placed in a holding yard (dense transit storage), commercial blueberry pollination fields and commercial sunflower seed production fields. *P* values indicate the statistical difference with the NF control at each time-point. Treatment groups were Diet A (nutritionally complete), Diet D (an internal reference diet), COMP (commercially available patty) and NF (no supplementary feed provided). *X* axis = time. (*a*) Average number of frames of bees per hive over time. (*b*) Average number of 5 cm^2^ areas of capped brood over time. Vertical bars below the *X*-axis indicate feeding events.

Over the 4.3-month period of Experiment 2, the colonies that were fed Diets A and D grew from *ca* 3.0 frames of bees to an average of 10.2 and 8.9 frames of bees, respectively (figure 3*a*). The COMP and NF colonies reached about half this size (average of 5.6 and 4.4 frames of bees, respectively).

Until day 41, no significant differences in changes from the start were observed between the diets (D, A and COMP) and the NF group. For Diet A, a significant treatment effect versus the NF group was observed from D68 onwards: the estimated number of frames of bees was on average 3.0 frames of bees higher than the NF colonies on D68 (95% CI: 0.29, 5.70, *P* = 0.030). This difference increased to 4.6 frames of bees on D106 (95% CI: 1.87, 7.29, *P* = 0.001) and 6.2 frames of bees on D133 (95% CI: 3.53, 8.94, *P* < 0.001). From D106 onwards Diet D colonies also showed a statistically significant increase in the estimated number of frames of bees compared with the NF group: on average 3.3 frames of bees higher for D106 (95% CI: 0.68, 5.88, *P* = 0.014) and 4.4 higher for D133 (95% CI: 1.78, 6.98, *P* = 0.001). In contrast, COMP colonies did not statistically differ in frames of bees from the NF group across time (*P* > 0.05).

On D133, 73% (8/11) of the Diet A colonies and 69% (9/13) of the Diet D colonies were larger than 6 frames of bees compared with 31% (4/13) in the COMP colonies and 24% (3/12) in the NF group. Colonies smaller than 6 frames of bees are unlikely to overwinter successfully and are considered too small to serve in pollination services.

Diet A and D colonies produced a higher amount of capped brood compared with COMP feed and NF group (figure 3*b*). An LMEM was fitted to the capped brood count data (see §2 and electronic supplementary material). In the period when the hives were in blueberry fields, the amount of capped brood was statistically similar across the four treatments and decreasing, consistent with beekeepers’ experiences that colonies declined in blueberry fields. However, as soon as the hives were out of blueberry fields, Diet A and Diet D colonies immediately and steadily increased brood production from D68 until D106 (the end of August). They showed a reduced capped brood area at the final assessment on D133 (22 September), consistent with the natural annual colony cycle whereby queens reduce egg-laying in the autumn. COMP colonies did not statistically differ in capped brood area from the NF group across time (*P* > 0.05).

NF and COMP hives continued to show reducing capped brood numbers until D68 and only showed a recovery in capped brood area on D106. COMP and NF had about half the brood present D68, D106 and D133 compared with Diets A and D. On D68, the Diet A and Diet D colonies had an average of 60 cm^2^ and 59 5 cm^2^ areas of capped brood, versus 22 for COMP and 29 for NF colonies. On D106 this was 100 for the Diet A colonies and 97 for the Diet D colonies, versus 53 for COMP and 40 for NF colonies. On D133, this was 75 for Diet A and 60 for Diet D, versus 42 for COMP and 37 for NF.

Diet A and Diet D colonies had a mortality of 9% (1/11 hives) and 8% (1/13 hives), respectively compared with 23% (3/13) for COMP colonies and 50% (6/12) for NF colonies by D133 (see electronic supplementary material).

## 4. Discussion and conclusion

In a classical nutrient depletion approach, we tested the hypothesis of whether 24-methylenecholesterol and isofucosterol are essential nutrients in the honeybee diet that cannot be fully replaced by the other five major honeybee phytosterols. This study presents the first demonstration of a pollen-free, nutritionally complete diet for honeybees (Diet A). We demonstrate that Diet A supports robust colony growth and health, even under the challenging conditions of commercial pollination operations. Surprisingly, our depletion experiments reveal that 24-methylenecholesterol, while abundant in honeybees, is not essential for brood production or viability. In contrast, we identify isofucosterol as a critical and previously unrecognized essential nutrient for honeybees. Building from flight cages to real-world pollination events, we demonstrate the importance of providing complete supplemental diet to full-size commercial colonies.

### (a) Diet A is a nutritionally complete artificial pollen-like diet

This artificial diet, Diet A (electronic supplementary material, table S1), contains the six major pollen and honeybee sterols (24-methylenecholesterol, isofucosterol, campesterol, stigmasterol, beta-sitosterol and cholesterol) at total concentrations and compositions in the range of nutritionally adequate pollen. In controlled flight cage studies (Experiment 1), Diet A supported stable brood production for over 108 days, equivalent to two full worker bee replacements [32–34] and 3−4 turnovers of 24-methylenecholesterol within adult bees [17] (electronic supplementary material, material S4). The sterol concentration and sterol profile in the honeybees fed Diet A is within the range of that of healthy colonies foraging in nature and was stable over the experiment (figure 2) (electronic supplementary material, table S4).

Importantly, the feed conversion ratio of Diet A was comparable to that of natural pollen, with 196 mg of Diet A required to produce one capped brood cell, similar to the 180 mg required with natural pollen [35–38] (electronic supplementary material, table S3). This demonstrates that Diet A is a nutritionally complete pollen-free but pollen-like diet for honeybee colonies and can effectively sustain colony growth and development, even under the various stressors imposed by the experimental conditions. This demonstrates a significant milestone toward a complete diet and allows for precisely designed restriction diets in future studies to further explore individual components of honeybee dietary needs.

### (b) Importance of 24-methylene-cholesterol

Contrary to expectations, our study reveals that 24MC, while abundant in honeybees, may not be as essential as previously thought. Depletion of 24MC from the diet (Diet C) resulted in a 14-fold reduction in its levels in honeybee tissues (2% of total sterols compared with 28% in bees fed the complete diet). However, the 24MC depletion had only a minor impact on brood production (not statistically significant, *p*‐value = 0.106). Furthermore, 24MC depletion resulted in only minor behavioural changes (reduced responsiveness).

The impact on the colony of a diet depleted in 24MC is much less severe (electronic supplementary material, figure S4 and table S2) than the impact of a 24EC-depleted diet (see next section). This lesser biological impact of 24MC depletion is unlikely to be owing to a better retention of 24MC in the sterol pool of the colony through the selective transfer of 24MC via worker jelly described in [11,14] and described in the next paragraph, since the isofucosterol levels were reduced 2.75-fold versus 14-fold for 24MC in the honeybees fed the respective depletion diets compared with the six-sterol diet.

We did find 24MC enriched by *ca* 10% in pupae/emerging adults versus nurse bees in colonies fed Diet A (32% of total sterols versus 28–29%, respectively). This is consistent with the reported ability of nurse bees to selectively transfer 24MC to brood via worker jelly [16,24], when 24MC is available.

Interestingly, 14 out of 21 newly emerged bees raised on the 24MC-depleted diet had no detectable 24MC. This suggests that 24MC is not strictly required for producing viable eggs, brood, or even effective worker jelly, despite being a major component of the latter [5,36]. This finding challenges the prevailing view of 24MC as the most critical sterol in honeybees.

While 24MC may not be essential for individual bee development, its depletion does have some effect at the colony level. The observed decline in brood production after 108 days, albeit minor, suggests a role for 24MC in overall colony health and productivity. Further investigation is needed to fully elucidate the complex roles of 24MC in honeybee physiology.

### (c) Isofucosterol is an essential sterol for honeybees

This study provides the first evidence that isofucosterol is an essential nutrient for honeybees. Depletion of isofucosterol from the diet resulted in severe effects on both brood production and neuromuscular function. This highlights isofucosterol’s critical role in honeybee health and demonstrates that it cannot be effectively replaced by the other five major sterols.

The observed neuromuscular impairments associated with isofucosterol deficiency (electronic supplementary material, table S2) likely disrupt essential colony tasks such as flight, foraging, pollination and brood care. While the mechanisms underlying this remain unclear, they may involve isofucosterol’s influence on cell membrane fluidity, protein raft formation or specific interactions with membrane proteins [39,40]. A sterol depletion experiment in *Drosophila* showed that when sterols are limited in the diet, they are selectively retained in the mushroom body and axonal membranes, consistent with sterols being more essential for neuronal function [41]. Further research is needed to elucidate these mechanisms.

After feeding the 24MC-depleted diet for 84−134 days, 24MC is near fully depleted in the honeybee tissues and isofucosterol is only partially depleted from the honeybee tissues after feeding the isofucosterol-depleted diet. Yet the brood production is much more affected by the isofucosterol deficiency: in nurse bees sampled on day 84 after feeding the isofucosterol-deficient diet, the share of isofucosterol was reduced by two-thirds (from 11% to 4% isofucosterol of total sterols) compared with nurse bees from colonies that received the diet with all six sterols. Comparing this with the 14-fold 24-methylenecholesterol reduction in the 24MC-depleted treatment, the data suggest that honeybees retain isofucosterol in the colony more efficiently and for longer than 24MC and hence appear to manage isofucosterol actively and conservatively.

A candidate for the isofucosterol retention mechanism is higher selective absorption or resorption of isofucosterol in the honeybee intestine. In an experiment designed to test the hypothesis that 24MC is selectively enriched in honeybees by selective intestinal resorption, Feldlaufer and Harrison [42] compared the sterol profile of pollen fed to honeybees with the sterol profile of the workers and the faeces they produced. Their data showed that 24MC, stigmasterol and campesterol proportions were significantly higher in faeces compared with worker bee tissues and that the percentage of isofucosterol of total sterols is reduced by 40% in faeces compared with workers [42], indicating a higher absorption of isofucosterol from the intestine versus the other sterols.

van der Vorst *et al*. [18] measured sterol concentrations daily in honeybee larvae between day 1 and day 5 of development. The percentage of 24MC and stigmasterol of total sterols remained constant between D1 and D5 at resp. approximately 70 and 9%. However, the percentage of isofucosterol of total sterols increased from 7.7 to 14.4%. This shows that selectively more isofucosterol than 24MC and stigmasterol is absorbed from the worker jelly and pollen consumed. Buttstedt *et al*. [24] report sterol concentrations in worker jelly (24MC 0.18 mg g^–1^; campesterol 0.05 mg g^–1^, beta-sitosterol 0.03 mg g^–1^, isofucosterol 0.02 mg g^–1^) and from their values isofucosterol makes up about 7% of total sterols in worker jelly, which is consistent with the 7.7% isofucosterol of total sterols in D1 larvae reported by van der Vorst *et al*. [18].

We observed that in newly emerging bees isofucosterol makes up a lower share of the total sterols than in adults: in pupae and adults emerged from capped brood collected from colonies after 134 days on Diet A, isofucosterol makes up 5% of total sterols, compared with 10–11% in the nurse bees and 15% in the diet (electronic supplementary material, table S4).

Our findings underscore the complex and nuanced roles of different sterols in honeybee physiology. Isofucosterol’s essentiality and the intricate mechanisms for its regulation highlight the need for a comprehensive understanding of honeybee nutritional requirements to ensure their health and productivity.

### (d) Implications for sustainable management of honeybee colonies

Beekeepers have long utilized protein supplements to stimulate brood production early in the season and to extend production late in the season. Feeding supplementary protein during the pollination season is less common than during the early or late seasons, as traditionally, nutritional needs are provided by the landscape. Despite this traditional reliance on natural forage, beekeepers are now reporting elevated and unsustainable summer colony losses [43]. These losses are likely driven by increasing nutritional stress on honeybee colonies, caused by factors such as habitat loss, climate change and farming practices. To assess the efficacy of Diet A under real-world conditions over an extended period, we conducted field trials with commercial honeybee colonies (Experiment 2). Colonies supplemented with Diet A thrived from May to September, navigating the challenges of commercial pollination, including transport, high-density stocking rates and periods of natural pollen scarcity. Sustained supplementary feeding of Diet A resulted in significantly stronger colonies with increased bee populations, better preparing them for winter survival, as larger colony size is a key predictor of overwintering success [44]. These findings underscore the potential of Diet A as a valuable tool for beekeepers to mitigate the nutritional challenges faced by honeybee colonies in modern agricultural landscapes.

Our findings have significant implications for the sustainable management of honeybee colonies. As demonstrated, honeybees require a complex array of sterols with distinct and partially overlapping roles. While 24MC, long considered the most critical sterol, may be less essential than previously thought, isofucosterol emerged as a key nutrient with a profound impact on colony growth and honeybee health. This nuanced understanding of honeybee nutritional needs underscores the importance of providing balanced and complete diets, especially in the face of increasing environmental challenges [45,46].

Many factors contribute to the nutritional stress experienced by honeybee colonies today, including habitat loss, pesticide use [47–49], climate change and monoculture agriculture [50,51]. These factors limit access to diverse and nutritious pollen sources, leading to colony declines and increased vulnerability to disease. Commercial beekeeping practices, while essential for crop pollination, can further exacerbate these challenges by exposing colonies to long-distance transport [52], high-density storage [47,53,54] and nutritionally poor forage.

Current artificial diets fail to fully meet the complex nutritional needs of honeybees [55], as seen in the commercial diet control groups. However, our development of Diet A, a complete pollen-free diet capable of sustaining colony growth and health even under challenging conditions, offers a promising solution. By providing beekeepers with a reliable tool to supplement natural forage and mitigate nutritional deficiencies, a complete supplemental diet can contribute to more sustainable beekeeping practices and improve colony resilience. Ultimately, healthier and more robust colonies translate to better pollination services, increased crop yields and a more secure food supply [50,56].

## Data Availability

Data supporting this paper are available in Dryad Digital Repository [57]. Supplementary material is available online [58].

## References

[1] Klein AM, Vaissière BE, Cane JH, Steffan-Dewenter I, Cunningham SA, Kremen C, Tscharntke T. 2007 Importance of pollinators in changing landscapes for world crops. Proc. R. Soc. B **274**, 303–313. (10.1098/rspb.2006.3721) PMC1702377 17164193

[2] Aizen MA, Garibaldi LA, Cunningham SA, Klein AM. 2008 Long-term global trends in crop yield and production reveal no current pollination shortage but increasing pollinator dependency. Curr. Biol. **18**, 1572–1575. (10.1016/j.cub.2008.08.066) 18926704

[3] Potts S, Ngo H, Biesmeijer J, Breeze T, Dicks L, Garibaldi L, Hill R, Settele J, Vanbergen A. 2016 The assessment report of the Intergovernmental Science-Policy Platform on Biodiversity and Ecosystem Services on pollinators, pollination and food production. IPBES ISBN 978-92-807-3567-3, Secretariat of the Intergovernmental Science-Policy Platform on Biodiversity and Ecosystem Services (IPEBES). See https://nora.nerc.ac.uk/id/eprint/519227.

[4] Degrandi-Hoffman G, Graham H, Ahumada F, Smart M, Ziolkowski N. 2019 The economics of honey bee (Hymenoptera: Apidae) management and overwintering strategies for colonies used to pollinate almonds. J. Econ. Entomol. **112**, 2524–2533. (10.1093/jee/toz213) 31504631

[5] Vaudo AD, Tooker JF, Grozinger CM, Patch HM. 2015 Bee nutrition and floral resource restoration. Curr. Opin. Insect Sci. **10**, 133–141. (10.1016/j.cois.2015.05.008) 29588000

[6] Furse S, Koch H, Wright GA, Stevenson PC. 2023 Sterol and lipid metabolism in bees. Metabolomics **19**, 78. (10.1007/s11306-023-02039-1) 37644282 PMC10465395

[7] Svoboda J, Feldlaufer M, Weirich G. 1994 Evolutionary aspects of steroid utilization in insects. J. Am. Chem. Soc. **562**, 126–139.

[8] Svoboda JA, Herbert EW, Thompson MJ, Shimanuki H. 1981 The fate of radiolabelled C_28_ and C_29_ phytosterols in the honey bee. J. Insect Physiol. **27**, 183–188. (10.1016/0022-1910(81)90126-8)

[9] Leponiemi M, Freitak D, Moreno-Torres M, Pferschy-Wenzig EM, Becker-Scarpitta A, Tiusanen M, Vesterinen EJ, Wirta H. 2023 Honeybees’ foraging choices for nectar and pollen revealed by DNA metabarcoding. Sci. Rep. **13**, 14753. (10.1038/s41598-023-42102-4) 37679501 PMC10484984

[10] McLellan AR. 1976 Factors affecting pollen harvesting by the honeybee. J. Appl. Ecol. **13**, 801–811. (10.2307/2402256)

[11] Coffey MF, Breen J. 1997 Seasonal variation in pollen and nectar sources of honey bees in Ireland. J. Apic. Res. **36**, 63–76. (10.1080/00218839.1997.11100932)

[12] Dimou M, Thrasyvoulou A. 2007 Seasonal variation in vegetation and pollen collected by honeybees in Thessaloniki, Greece. Grana **46**, 292–299. (10.1080/00173130701760718)

[13] de Vere N, Jones LE, Gilmore T, Moscrop J, Lowe A, Smith D, Hegarty MJ, Creer S, Ford CR. 2017 Using DNA metabarcoding to investigate honey bee foraging reveals limited flower use despite high floral availability. Sci. Rep. **7**, 42838. (10.1038/srep42838) 28205632 PMC5311969

[14] Brodschneider R, Crailsheim K. 2010 Nutrition and health in honey bees. Apidologie **41**, 278–294. (10.1051/apido/2010012)

[15] Zu P *et al*. 2021 Pollen sterols are associated with phylogeny and environment but not with pollinator guilds. New Phytol. **230**, 1169–1184. (10.1111/nph.17227) 33484583 PMC8653887

[16] Svoboda JA, Herbert EW, Lusby WR, Thompson MJ. 1983 Comparison of sterols of pollens, honeybee workers, and prepupae from field sites. Arch. Insect Biochem. Physiol. **1**, 25–31. (10.1002/arch.940010104)

[17] Gage SL, Calle S, Jacobson N, Carroll M, DeGrandi-Hoffman G. 2020 Pollen alters amino acid levels in the honey bee brain and this relationship changes with age and parasitic stress. Front. Neurosci. **14**, 231. (10.3389/fnins.2020.00231) 32265638 PMC7105889

[18] van der Vorst E, Mattys J, Jacobs FJ, de Rycke PH. 1983 Fatty acid and sterol composition during larval development in the honeybee. J. Apic. Res. **22**, 3–8. (10.1080/00218839.1983.11100552)

[19] Herbert EW, Svoboda JA, Thompson MJ, Shimanuki H. 1980 Sterol utilization in honey bees fed a synthetic diet: effects on brood rearing. J. Insect Physiol. **26**, 287–289. (10.1016/0022-1910(80)90135-3)

[20] Tian W *et al*. 2018 Architecture of the native major royal jelly protein 1 oligomer. Nat. Commun. **9**, 3373. (10.1038/s41467-018-05619-1) 30135511 PMC6105727

[21] Kucharski R, Maleszka R, Hayward DC, Ball EE. 1998 A royal jelly protein is expressed in a subset of Kenyon cells in the mushroom bodies of the honey bee brain. Naturwissenschaften **85**, 343–346. (10.1007/s001140050512) 9722965

[22] Hojo M, Kagami T, Sasaki T, Nakamura J, Sasaki M. 2010 Reduced expression of major royal jelly protein 1 gene in the mushroom bodies of worker honeybees with reduced learning ability. Apidologie **41**, 194–202. (10.1051/apido/2009075)

[23] Chakrabarti P, Lucas HM, Sagili RR. 2020 Evaluating effects of a critical micronutrient (24-methylenecholesterol) on honey bee physiology. Ann. Entomol. Soc. Am. **113**, 176–182. (10.1093/aesa/saz067) 32410742 PMC7212396

[24] Buttstedt A, Pirk CWW, Yusuf AA. 2023 Mandibular glands secrete 24-methylenecholesterol into honey bee (Apis mellifera) food jelly. Insect Biochem. Mol. Biol. **161**, 104011. (10.1016/j.ibmb.2023.104011) 37716535

[25] Chakrabarti P, Lucas HM, Sagili RR. 2020 Novel insights into dietary phytosterol utilization and its fate in honey bees (Apis mellifera L.). Molecules **25**, 571. (10.3390/molecules25030571) 32012964 PMC7036750

[26] Wright GA, Nicolson SW, Shafir S. 2018 Nutritional physiology and ecology of honey bees. Annu. Rev. Entomol. **63**, 327–344. (10.1146/annurev-ento-020117-043423) 29029590

[27] Tsuruda JM, Chakrabarti P, Sagili RR. 2021 Honey bee nutrition. Vet. Clin. N. Am **37**, 505–519. (10.1016/j.cvfa.2021.06.006) 34689917

[28] Furse S *et al*. 2024 Systemic analysis of lipid metabolism from individuals to multi-organism systems. Mol. Omics **20**, 570–583. (10.1039/d4mo00083h) 39246063 PMC11381968

[29] Furse S, Fernandez-Twinn DS, Jenkins B, Meek CL, Williams HEL, Smith GCS, Charnock-Jones DS, Ozanne SE, Koulman A. 2020 A high-throughput platform for detailed lipidomic analysis of a range of mouse and human tissues. Anal. Bioanal. Chem. **412**, 2851–2862. (10.1007/s00216-020-02511-0) 32144454 PMC7196091

[30] R Core Team. 2023 R: A language and environment for statistical computing. See https://www.R-project.org.

[31] Bates D, Mächler M, Bolker B, Walker S. 2015 Fitting linear mixed-effects models using lme4. J. Stat. Softw. **67**, 48. (10.18637/jss.v067.i01)

[32] Free J, Spencer-Booth Y. 1959 The longevity of worker honey bees (Apis mellifera). Proc. R. Entomol. Soc **34**, 141–150. (10.1111/j.1365-3032.1959.tb00230.x)

[33] Fukuda H, Sekiguchi K. 1966 Seasonal change of the honeybee worker longevity in Sapporo, North Japan, with notes on some factors affecting lifespan. Jpn. J. Ecol **16**, 206–212. (10.18960/seitai.16.5_206)

[34] Remolina SC, Hughes KA. 2008 Evolution and mechanisms of long life and high fertility in queen honey bees. AGE **30**, 177–185. (10.1007/s11357-008-9061-4) 19424867 PMC2527632

[35] Crailsheim K, Schneider LHW, Hrassnigg N, Bühlmann G, Brosch U, Gmeinbauer R, Schöffmann B. 1992 Pollen consumption and utilization in worker honeybees (Apis mellifera carnica): dependence on individual age and function. J. Insect Physiol. **38**, 409–419. (10.1016/0022-1910(92)90117-v)

[36] Wille H, Imdorf A. 1983 Stickstoffversorgung des Bienenvolkes. Allg. Dtsch. Imkerztg. **2**, 37–50.

[37] Keller I, Fluri P, Imdorf A. 2005 Pollen nutrition and colony development in honey bees: part 1. Bee World **86**, 3–10. (10.1080/0005772x.2005.11099641)

[38] Keller I, Fluri P, Imdorf A. 2005 Pollen nutrition and colony development in honey bees—part II. Bee World **86**, 27–34. (10.1080/0005772x.2005.11099650)

[39] Jing X, Behmer ST. 2020 Insect sterol nutrition: physiological mechanisms, ecology, and applications. Annu. Rev. Entomol. **65**, 251–271. (10.1146/annurev-ento-011019-025017) 31600456

[40] Fantini J, Barrantes F. 2009 Sphingolipid/cholesterol regulation of neurotransmitter receptor conformation and function. Biochim Biophys Acta Biomembr **1788**, 2345–2361. (10.1016/j.bbamem.2009.08.016) 19733149

[41] Carvalho M *et al*. 2010 Survival strategies of a sterol auxotroph. Development **137**, 3675–3685. (10.1242/dev.044560) 20940226 PMC2964098

[42] Feldlaufer MF, Harrison DJ. 2020 Neutral sterols in honey bee (Apis mellifera) feces. J. Apic. Res. **59**, 1033–1036. (10.1080/00218839.2020.1753917)

[43] Aurell D, Bruckner S, Wilson M, Steinhauer N, Williams GR. 2024 A national survey of managed honey bee colony losses in the USA: results from the Bee Informed Partnership for 2020–21 and 2021–22. J. Apic. Res. **63**, 1–14. (10.1080/00218839.2023.2264601)

[44] Döke MA, McGrady CM, Otieno M, Grozinger CM, Frazier M. 2019 Colony size, rather than geographic origin of stocks, predicts overwintering success in honey bees (Hymenoptera: Apidae) in the Northeastern United States. J. Econ. Entomol. **112**, 525–533. (10.1093/jee/toy377) 30566679

[45] Insolia L, Molinari R, Rogers SR, Williams GR, Chiaromonte F, Calovi M. 2022 Honey bee colony loss linked to parasites, pesticides and extreme weather across the United States. Sci. Rep. **12**, 20787. (10.1038/s41598-022-24946-4) 36456591 PMC9714769

[46] O’Neal ST, Anderson TD, Wu-Smart JY. 2018 Interactions between pesticides and pathogen susceptibility in honey bees. Curr. Opin. Insect Sci. **26**, 57–62. (10.1016/j.cois.2018.01.006) 29764661

[47] Steinhauer N, Kulhanek K, Antúnez K, Human H, Chantawannakul P, Chauzat MP, vanEngelsdorp D. 2018 Drivers of colony losses. Curr. Opin. Insect Sci. **26**, 142–148. (10.1016/j.cois.2018.02.004) 29764654

[48] Schmickl T, Crailsheim K. 2004 Inner nest homeostasis in a changing environment with special emphasis on honey bee brood nursing and pollen supply. Apidologie **35**, 249–263. (10.1051/apido:2004019)

[49] Stoner KA, Hendriksma HP, Tosi S. 2023 Editorial: Pollen as food for bees: diversity, nutrition, and contamination. Front. Sustain. Food Syst. **6**, 1129358. (10.3389/fsufs.2022.1129358)

[50] Thebeau JM *et al*. 2023 Are fungicides a driver of European foulbrood disease in honey bee colonies pollinating blueberries? Front. Ecol. Evol. **11**, 1073775. (10.3389/fevo.2023.1073775)

[51] Noordyke ER, van Santen E, Ellis JD. 2022 Evaluating the strength of western honey bee (Apis mellifera L.) colonies fed pollen substitutes over winter. J. Appl. Entomol. **146**, 291–300. (10.1111/jen.12957)

[52] DeGrandi-Hoffman G, Chen Y, Rivera R, Carroll M, Chambers M, Hidalgo G, de Jong EW. 2016 Honey bee colonies provided with natural forage have lower pathogen loads and higher overwinter survival than those fed protein supplements. Apidologie **47**, 186–196. (10.1007/s13592-015-0386-6)

[53] Goodrich BK, Goodhue RE. 2020 Are all colonies created equal? The role of honey bee colony strength in almond pollination contracts. Ecol. Econ. **177**, 106744. (10.1016/j.ecolecon.2020.106744)

[54] Crailsheim K. 1998 Trophallactic interactions in the adult honeybee (Apis mellifera L.). Apidologie **29**, 97–112. (10.1051/apido:19980106)

[55] Wright GA, Nicholson SW, Shafir S. 2018 Nutritional physiology and ecology of honey bees. Annu. Rev. Entomol. **63**, 327–334. (10.1146/annurev-ento-020117-043423) 29029590

[56] Brodschneider R, Omar E, Crailsheim K. 2022 Flight performance of pollen starved honey bees and incomplete compensation through ingestion after early life pollen deprivation. Front. Physiol. **13**, 1004150. (10.3389/fphys.2022.1004150) 36569746 PMC9780383

[57] Bogaert T, Reams T, Maillet I, Kulhanek K, Duyck M, Eertmans F, Fauvel AM, Hopkins B, Bogaert J. 2024 Data from: A nutritionally complete pollen-replacing diet protects honeybee colonies during stressful commercial pollination—requirement for isofucosterol. Dryad Digital Repository. (10.5061/dryad.wpzgmsc08) PMC12000826 40235288

[58] Bogaert T, Reams T, Maillet I, Kulhanek K, Duyck M, Eertmans F *et al*. 2025 Supplementary material from: A nutritionally complete pollen-replacing diet protects honey bee colonies during stressful commercial pollination - Requirement for isofucosterol. Figshare. (10.6084/m9.figshare.c.7736315) PMC12000826 40235288

